# Testing the impact of interpersonal regulatory fit on empathy, helping intentions, and prosocial behaviour

**DOI:** 10.1371/journal.pone.0270462

**Published:** 2022-07-18

**Authors:** Rinad Bakhti, Karl-Andrew Woltin, Kai Sassenberg, John Rae

**Affiliations:** 1 Department of Psychology, University of Roehampton, London, United Kingdom; 2 Department of Psychology, Catholic University of Louvain, Louvain‐la‐Neuve, Belgium; 3 Leibniz-Institut für Wissensmedien, Tübingen, Germany; 4 University of Tübingen, Tübingen, Germany; Public Library of Science, UNITED STATES

## Abstract

Similarity with others in need regarding various attributes [e.g., social group membership] has been shown to increase individuals’ empathic responses, willingness to help and prosocial behaviour. We tested whether a subtle similarity, namely of observers’ and targets’ self-regulatory orientation in terms of a promotion or prevention regulatory focus [i.e., interpersonal regulatory fit], would entail similar effects. Interpersonal regulatory [mis]fit was conveyed through focus-congruent or -incongruent emotional reactions which targets, facing distressing situations, expressed. We predicted that when observer participants’ regulatory focus fits with targets’ negative emotional reaction [i.e., promotion focus—dejection or prevention focus—agitation], they would be more likely to express empathy, willingness to help, and to engage in prosocial behaviour towards this target compared to conditions of misfit. Five studies relied on observers’ chronic regulatory focus [Study 1, 3, & 4] and situationally induced regulatory focus [Study 2 & 5] and presented different distressing scenarios with targets conveying focus [in]congruent negative emotions. Inconsistent results emerged across the studies, which indicated misfit, fit and no effects. Study characteristics did not suggest a moderator explaining these inconsistent findings. An internal meta-analysis across all studies indicated that overall there was no evidence of either a fit or a misfit effect. This work sheds light on the technical challenges of exploring relations between subtle interpersonal regulatory [mis]fit and prosocial reactions. Implications for future research are discussed, including the importance of creating stronger interpersonal [mis]fit experiences by means of incorporating descriptions of distressed targets’ hindered goal pursuits as well as negative reactions.

## Introduction

When seeing others in distress, people are more likely to help and respond empathetically if they perceive similarities between themselves and the distressed person. This has been attested by several studies considering the similarity between observers and distressed targets regarding various attributes and characteristics [e.g., [1–3]. These characteristics are wide-ranging and include, but are not limited to, social group membership, similar interests, values and similar past experiences [4–6]. For example, participants are more likely to offer financial help after natural disasters to people of the same nationality [7]. Also, people with past experiences of loss of a parent or pet report greater empathy towards targets who describe going through similar experiences [4]. It is notable that these effects occurred when participants were only aware of one aspect that they shared with the target person, without having much further information about them or their circumstances. Consequently, differences in empathy, helping intentions and prosocial behaviours can, at least in part, be ascribed to perceived similarities regarding only the shared characteristic in question.

The current work seeks to add to the emerging literature on similarity having a positive impact on empathy and prosocial responses by considering a novel attribute, namely similarity in terms of observers’ regulatory focus and targets’ focus-congruent emotional response to their distressing situation: interpersonal regulatory fit [8–11]. Unlike group membership or other previously studied characteristics, interpersonal regulatory fit does not require conscious awareness and regulatory focus is a concept individuals are not likely to be explicitly aware of, but might have an implicit notion of [12]. In what follows we first review literature suggesting that under conditions of interpersonal regulatory fit, observers and targets are likely to appraise situations similarly and express empathy. We then briefly review regulatory focus theory and summarise previous work on the beneficial effects of interpersonal regulatory fit, which jointly informed our hypothesis.

### The appraisal theory of empathy

Empathy is conceived of as an internal and emotional response towards, and elicited by, another person, which is congruent with the other’s welfare [13–15]. Empathy is conceptualized as affective in nature and can entail feelings such as sympathy, compassion, and softheartedness *for* someone else [13, 16]. Furthermore, in much of the literature, empathy is assumed to contribute to prosocial behaviour [17–19] which refers to voluntarily performed actions aimed at increasing the welfare of another person or people, including actions such as helping, sharing, cooperating, and comforting [20]. Importantly, the appraisal theory of empathy proposes that based on the appraisal of a given situation that someone else is experiencing [i.e., how one evaluates or interprets the situation], second-hand emotions towards others are formed, just as first-hand emotions are based on how one evaluates situations oneself is experiencing [21]. That is, an appraisal of a target’s situation determines the emotional experience that an observer would have if they would find themselves in the same situation. If an observer’s appraisal of a situation is congruent with that of a target, this will result in observers feeling empathy. However, if an observer appraises the situation differently, this will result in observers feeling either a different emotion from empathy or showing a relatively unemotional reaction. Since past experiences influence how people appraise their own and others’ situations, this appraisal perspective explains why people are especially empathetic with others when they themselves have experienced similar situations in the past [e.g., [5]]. In essence, the theory proposes that the interpretation of events is personal and subjective, and that the same objective attributes of a situation are appraised and perceived similarly or differently depending on attributes that observers and targets share or do not share. A variable that nicely lends itself to study these propositions, because it is known to influence interpersonal similarities and differences in terms of how events are interpreted an appraised, is regulatory focus [22], which we address now.

### Regulatory focus theory

According to regulatory focus theory, two systems of self-regulation operate within individuals [8, 22]. Nurturance-driven self-regulation, called promotion focus, is concerned with ensuring advancement, accomplishment, and growth [ideals], largely using eager means to these ends. Conversely, security-driven self-regulation, called prevention focus, is concerned with safety and the fulfillment of duties and obligations [oughts], largely using vigilant means to these ends. Positive and negative events are appraised in different ways by these two foci. Positive events are appraised as gains in a promotion focus but as non-losses in a prevention focus; whereas negative events are appraised as non-gains in a promotion focus but as losses in a prevention focus [23, 24]. Consequently, these different appraisals entail different emotional experiences [10, 25]. That is, promotion-focused individuals report dejection- [cheerfulness-] related emotions such as sadness after negative [positive] outcomes or events. By contrast, prevention-focused individuals report agitation- [quiescence]- related emotions such as anxiety [calmness] after negative [positive] outcomes or events [10, 25, 26]. Regulatory focus differs across individuals and can be situationally induced [23].

### Interpersonal regulatory fit

When two individuals share the same regulatory focus, they are likely to experience interpersonal regulatory fit, a feeling of “rightness” [27], which recent research has found to entail several positive consequences. For example, individuals are more likely to forgive another person who committed a transgression when the transgressor shares their regulatory focus [28]. Interpersonal fit has also been found to intensify liking [29] and in on-going relationships participants viewed partners as more instrumental in their own goal-pursuit when both shared a promotion focus [11].

In the leader-follower context, interpersonal fit between supervisors and followers likewise resulted in positive consequences, such as greater commitment and improved relationship quality with one’s supervisor [30], reduced turnover intentions of employees [31], and followers feeling more valued [32]. Furthermore, people evaluating applicants conveying their regulatory focus in application letters reported a greater perceived fit and likelihood of liking and hiring them under conditions of interpersonal fit [27]. Taken together, this research indicates that similarity in regulatory focus, as with other attributes, may help the observer perceive the target as more relevant to the self, allowing them to recognise aspects of themselves in the target person, leading to the benefits of interpersonal regulatory fit above.

Overall, interpersonal regulatory fit has thus been found to entail positive consequences in diverse domains. Building on this previous work, the current work considers the impact of regulatory fit on empathy, willingness to help and prosocial behaviour. More specifically it investigates whether under condition of fit between observers being confronted with targets expressing focus-congruent emotions [i.e., promotion focused observers learning about dejected targets or prevention focused observers learning about agitated targets] participants will report greater perceived fit with the person, willingness to help, empathy and prosocial behaviour.

Importantly, we note that different to emotional similarity, in interpersonal regulatory fit an observer does not experience the same emotion as a target. Rather, based on their presumably similar appraisal of the situation [e.g., based on their gain/loss appraisal], observers can be expected to assume they would experience the same or a very similar emotion *if* they were faced with the given situation, and thus to deem the emotion expressed by the target as more ‘fitting’ [right, adequate etc.; 21]. Indeed, differences in one’s regulatory focus are expected to influence one’s appraisal of situations [22]. In order to ensure that the effects of interpersonal regulatory fit go beyond possible effects of mere similarity, the current work will take into account such potential resemblance [“How much does the person in the text resemble you?”; 29].

To the best of our knowledge, the only work investigating a similar question focused on self-discrepancies. According to self-discrepancy theory [33], a self-discrepancy occurs when the actual self [which includes the attributes one currently possesses] does not match either the ideal or ought self [attributes one would ideally like to possess or attributes one thinks one ought to possess, respectively]. In this study, participants’ self-discrepancies were assessed and they were then presented with targets describing a distressing incident as a result of shyness and expressing either dejection or agitated-related emotions; sharing a self-discrepancy with targets increased observers’ perceived similarity and enhanced empathic responses [34]. However, apart from being concerned with self-discrepancies rather than regulatory focus, which is a new and different theory [35], Houston included only participants with extreme scores on self-discrepancy measures [i.e., participants were pre-selected to have either very high or very low ideal and ought self-discrepancies]. Contrary, the present work considers normal distributions of participants’ promotion and prevention foci based on a regulatory focus measure. Moreover, to test for causal evidence, the current work also induces regulatory focus in several studies. Furthermore, the present work goes beyond that of Houston in that in addition to empathy it considers willingness to help and prosocial behaviour. Finally, seeking to speak to generalizability of effects, various scenarios in diverse contexts are relied upon across the different studies. Overall, this research aims to establish interpersonal regulatory fit as an antecedent of empathy, willingness to help and prosocial behaviour.

### The current research

In the present studies, participants’ regulatory focus was measured or induced, and they were subsequently presented with a scenario [differing in content across studies] in which a distressed target expressed an emotional reaction of dejection or agitation in reaction to the situation conveyed in the scenario. Participants were told the scenario text had been allocated randomly and that it had been written by a participant in a previous qualitative study; they thus believed that the target response they read stemmed from a larger pool of responses. Participants were invited to indicate to what extent they felt empathy with the target, would be willing to help them, and to provide help. The current set of studies thus sought to test the hypothesis that interpersonal fit would increase empathy, willingness to help and prosocial behaviour, providing correlational [Study 1, 3, & 4] and experimental evidence [Study 2 & 5] across different distressing scenarios. Additionally, an internal meta-analysis of the studies was conducted to gauge the nature of effects across studies.

We assessed several control variables in order to ensure that potential effects of interpersonal regulatory fit could be explained as going beyond mere perceived similarity and/or other potentially relevant factors not of focal interest. Pilot testing of scenarios revealed that different emotional target reactions at times give rise to different perceptions of the situation, the intensity of the negative emotion expressed, or the similarity with the target. To be able to gauge the contribution of interpersonal regulatory fit beyond such–for this research–secondary factors, we systematically considered them, in line with previous work. For example, Hamstra et al. [27] report interpersonal regulatory fit effects even when accounting for–and thus as they stress *beyond–*similarity.

For all studies, sample sizes were determined using G*Power 3.1 [36] for small to medium size effects [Studies 1, 3, 4 f^2^ = 0.08; Studies 2 and 5, f = 0.2], with α = .05 and 90% power [1-β]. Participants completed all studies online, taking an average of 12 minutes, for which they were paid GBP 6 hourly wage. Participants were recruited through Prolific Academic [www.prolific.ac], a platform created for the purpose of recruiting research participants. Individuals who are signed up to this platform and meet the criteria for the study [above 18 years of age; spoke English as a first language] were able to take part. Participants from this and similar platforms have been shown to provide reliable responses [e.g., [37, 38]. The only exception to the above is Study 2, in which student participants took part online and were rewarded with course credit.

All data analyses were conducted on Statistical Package for the Social Sciences, Version 25. In studies relying on regression analyses, outliers with studentized deleted residuals SDR > |2.59| were removed, and in studies relying on ANOVA, outliers with studentized residuals greater than SRE > |2.59| were removed [39]. This resulted in the removal of participants as follows: In Study 1 *n* = 4 [1.3%], Study 2 *n* = 6 [2.4%], in Study 3 *n* = 3 [1%], in Study 4 *n* = 8 [3.2%], in Study 5 *n* = 7 [2%] [including the outliers in the analyses does not alter significance levels].

We report all manipulations and all measures in all of the studies as well as all exclusions [if any]. Data and analyses scripts for all studies as well as the mini meta-analysis can be accessed on the Open Science Framework [OSF; https://osf.io/3q5g2/?view_only=0c0b7a22c739425cac18f8772c9785ee].

## Study 1

The first study aimed to test our hypothesis that interpersonal regulatory fit increases willingness to help, empathy, and prosocial response as measured by the level of engagement in writing message of advice to a distressed person. Participants’ regulatory foci were measured before they were confronted with a scenario of a target in distress expressing either dejection or agitation. The scenario described a person struggling to adjust to a new and challenging work environment.

### Methods

#### Participants and design

All studies obtained ethical approval from the departmental ethics committee of the University of Roehampton where this research was conducted. Participants in all studies were informed that the respective study involved personal past experiences and reactions to distressed others. If they provided informed consent, they were only allowed to take part if English was their native language and if they were 18 years of age or older. A total of 303 participants took part in an experiment with three experimental conditions [target emotional reaction: dejection, agitation, no emotional reaction–control]. Participants’ regulatory focus was measured as an additional predictor variable. The dependent variables included perceived interpersonal regulatory fit, willingness to help, empathy, and level of engagement [as measured by time spent] in writing a message of advice to the person in the scenario.

Beside the exclusion criteria mentioned above, 115 participants were excluded because they failed a question probing their attention to instructions [*n* = 12; [40]] and/or a manipulation check probing if they correctly recalled the emotion expressed by the target [i.e., either dejection or agitation or no emotional reaction, *n* = 103]. All but 7 participants who failed the manipulation check were in the control condition and thus read about a target who had not expressed any emotional reaction. As such, the control condition was removed from all analyses.

The final sample thus comprised 179 participants [116 women, 62 men; *M*_age_ = 32.74, *SD*_age_ = 10.72], with 91 participants in the target dejection emotional reaction condition and 88 in the target agitation emotional reaction condition. A sensitivity analysis using G*Power [36] for this study based on the final sample size and with an α-level = .05 and a power of 90%, indicated that the minimum size of the effect that could reliably be detected is f^2^ = 0.081.

#### Materials and procedure

Participants filled in the Regulatory Focus Questionnaire [RFQ; [41]], an 11-item questionnaire containing a promotion focus subscale [6 items; α = .72; *M =* 3.34; *SD =* .66, e.g., “How often have you accomplished things that got you psyched to try even harder?”] and a prevention focus subscale [5 items; α = .85; *M =* 3.24, *SD =* .88, e.g., “Not being careful enough has gotten me into trouble at times”, reverse-scored]. They rated each item on a 5-point scale ranging from 1 [*never or seldom; certainly false*] to 5 [*very often; certainly true*]. In this and some other studies additional measures were assessed for exploratory purposes. A list of all measures and the order in which they were taken can be found in the supplemental material online on OSF [https://osf.io/3q5g2/?view_only=0c0b7a22c739425cac18f8772c9785ee].

Participants were then presented with a scenario of a person struggling to adjust to a new and challenging work environment [see S1 Appendix]. For example, the target stated that: “I do not feel as appreciated as in my previous job and I am feeling much less motivated as a result. Sometimes I think I should not have changed jobs, but it’s too late now.” The scenario was constructed with the aim that it would be equally plausible for the target to express dejection or agitation in response to the situation. In all conditions participants read the same scenario, which did not mention any emotional reaction of the protagonist. Only afterwards participants were randomly assigned to an emotional reaction of the target expressing either dejection or agitation [e.g., “I am actually feeling very sad [nervous] about this transition”; see S1 Appendix].

To measure interpersonal regulatory fit [α = .89; *M* = 4.92; *SD* = 1.02], participants subsequently reported their liking of the person [3 items; e.g., “I like the person”] and their feelings of rightness regarding the person [3 items; e.g., “I feel that the person and I would fit together well”]. These questions were based on items from Hamstra, Van Yperen, Wisse, and Sassenberg [42] and Righetti, Finkenauer, and Rusbult [11]. Participants responded on a 7-point scale [1 = *strongly disagree*, 7 = *strongly agree*].

Subsequently, they filled in a 4-item measure of empathy [i.e., to what extent they felt sympathetic, moved, compassionate, and warm towards the target; α = .83; *M* = 4.85; *SD* = 1.13], based on Batson et al. [13]. Responses were given on a 7-point scale [1 = *not at all*, 7 = *very much*]. Willingness to help was measured with one question: “To what extent would you be willing to help this person?” [*M* = 5.68; *SD* = 1.10; 1 = *not at all*, 7 = *very much*]. In order to include a behavioural measure of help, participants were asked to write a message of advice to the person in the scenario on how best to deal with their situation. The time spent on the message was used as an indicator to assess their level of engagement with this message in seconds [*M* = 248.43; *SD* = 498.41].

We assessed several variables in order to potentially control for the different emotional reactions of the target leading to different perceptions of the severity of the situation [“How severe did you find the situation of the person in the text?”; *M* = 4.07; *SD* = 1.37], of the intensity of the negative emotion [“How intense was the negative emotion the person was expressing in the text?”*; M* = 5.17; *SD* = 1.23], and of resemblance with the protagonist [“How much does the person in the text resemble you?”; *M* = 4.02; *SD* = 1.71]. Participants responded to each item on a 7-point scale [1 = *not at all*; 7 = *very much*]. Finally, participants’ current positive mood was assessed with 12 items [26], asking participants to rate, for example, how tense [reversed], relaxed, discouraged [reversed], and happy they felt on a 7-point scale [1 = *I do not at all feel like this*; 7 = *I very much feel like this*; α = .91; *M* = 4.60; *SD* = 1.18]. Finally, a manipulation check asked them to recall what kind of emotional reaction the target in the scenario they had read expressed [dejection or agitation or no emotional reaction].

### Results

#### Preliminary analyses

Each control variable [i.e., severity, intensity, resemblance, and current positive mood] was regressed on participants’ z-transformed promotion focus and prevention focus, the target reaction condition [coded 1 = dejection, -1 = agitation], the promotion by condition, prevention by condition and the promotion by prevention two-way interactions, as well as the promotion by prevention by condition three-way interaction. [Not including the promotion by prevention two-way and three-way interaction as predictors does not alter the results in this study, nor in Studies 3 and 4 with similar designs. The same z-standardization and condition coding is applied in Studies 3 and 4, which also rely on chronic regulatory focus].

A main effect of target reaction condition [*B* = .15, *SE* = .10, *t* = -2.02, *p* = .045, CI_95%_:.01,.42] indicated that in the dejection reaction condition the situation was perceived as more severe [*M* = 4.27, *SD* = 1.32] compared with the agitation reaction condition [*M* = 3.85, *SD* = 1.41]. A further main effect of target reaction condition [*B* = -.23, *SE* = .12, *t* = -3.23, *p* = .001, CI_95%_: -.62, -.16] indicated that in the agitation reaction condition the target was perceived as more similar [*M* = 4.32, *SD* = 1.73] compared with the dejection reaction condition [*M* = 3.74, *SD* = 1.64]. Additionally, participants with a stronger promotion focus were significantly less likely to perceive the target as similar [*B* = -.39, *SE* = .12, *t* = -5.59, *p* < .001, CI_95%_:-.91, -.44] and more likely to report higher positive mood [*B* = .46, *SE* = .08, *t* = 6.56, *p* < .001, CI_95%_: .36, .70]. No other significant effects emerged [all other *ts*< 1.81, all other *ps*>.07]. Given these unexpected effects, in the main analyses below we control for severity, resemblance and current positive mood.

#### Main analyses

Willingness to help, empathy, perceived fit, and time spent writing a message of advice to the protagonist were regressed separately on the same predictors as in the multiple regression above, while controlling for severity, resemblance and current positive mood. The stronger participants’ promotion focus was, the more they reported willingness to help [*B* = .25, *SE* = .09, *t* = 3.05, *p* = .003, CI_95%_: .13, .45]. No other significant effects emerged [all *ts*<1.84, all *ps*>.07]. [The main effect remained significant and no other effects emerged when not including the control variables].

### Discussion

Contrary to the hypothesis, this study found no significant effects of interpersonal regulatory fit between an observer and a person in distress displaying regulatory focus-congruent emotions on perceived fit, empathy, willingness to help or time spent on a message written to the person. Importantly, participants perceived the scenario resulting in the target expressing dejection as more severe than the exact same situation resulting in agitation. Such increased perceptions of severity may be due to reactions of dejection generally conveying a sense of loss, a lack of coping potential and suggesting that there is little hope for redress [43, 44]. Ideally, the distressing scenario should have produced similar levels of perceived severity, and also of resemblance in participants–independent of targets expressing dejection or agitation. In order to ensure this, pre-tests of the scenarios used in Studies 3, 4 and 5 were conducted [see S2 Appendix]. [Study 2 was run before data analysis for Study 1 was completed and thus the scenario was not pretested].

It is also noteworthy that the vast majority of participants in the control condition [93%] failed the manipulation check. Even though participants in the control group only read the descriptive situation scenario but not an emotional target reaction, the majority still reported that the target had conveyed a dejection or agitation emotional response [which was not the case in this condition]. It thus seems that participants nonetheless attributed an emotion to the protagonist. Accordingly, and given that a no-emotion control condition does not seem to serve its function, this control condition was dropped in subsequent studies.

## Study 2

Compared to Study 1, three changes were made. First, participants’ regulatory focus was manipulated rather than measured, because this might provide clearer evidence. To illustrate, given the presumed independence of the foci [22, 23] a participant high in promotion in Study 1 could also have been high in prevention, thus reducing the likelihood of experiencing fit with a dejected target. Second, participants were recruited from a student population rather than on Prolific Academic. Even though Study 1 ensured participants were attentive by means of an attention check, this change aimed to ensure the lack of effects in Study 1 did not stem from participants taking part for professional reasons. Third, a different scenario, was used. On the one hand, this change was implemented as the scenario in Study 1 might not have been suitable to test our hypothesis; on the other hand, the new scenario was targeted to the student participant population, Specifically, participants read about a student finding it difficult to adapt to university and expressing either dejection or agitation as a result.

### Methods

#### Participants and design

Participants were randomly assigned to conditions in a 3 [observer regulatory focus: promotion, prevention, control] x 2 [target emotional reaction: dejection, agitation] between-subjects design. The same dependent variables as in the previous study were assessed. A total of 245 participants from an undergraduate psychology student pool at a UK University completed the study for course credit. In addition to the exclusion criteria mentioned above, a total of 84 participants who failed the attention check and 49 participants who failed the manipulation check probing the kind of emotional reaction the person expressed in the scenario they had read [dejection vs. agitation], were excluded. The final sample comprised 132 participants [107 women, 25 men; *M*_age_ = 21.8, *SD*_age_ = 6.96]. A sensitivity analysis using G*Power [36] based on the final sample size and with an α-level = .05 and a power of 90%, indicated that the minimum size of the effect that could reliably be detected is f = 0.28.

#### Materials and procedure

The same procedure as in Study 1 was used with the following exceptions. First, rather than measuring regulatory focus, participants were exposed to either a promotion or prevention focus induction, or were not exposed to such an induction [i.e., the control condition; [41]]. In both the promotion and prevention focus induction conditions, participants were asked to report three past events, two entailing promotion or prevention success and one entailing promotion or prevention failure. For example, the promotion condition entailed reporting on “a time in the past when compared to most people you were able to get what you wanted out of life.” Similarly, the prevention condition entailed reporting on “a time in the past when you stopped yourself from acting in a way that others would consider unacceptable.” Participants in the control condition were simply asked to report on three recent past events unrelated to regulatory focus concerns [i.e., taking public transport, going to the supermarket, describing their most recent purchase].

Second, participants were subsequently presented with a scenario of a distressed student struggling to adjust to university life [see S3 Appendix]. For example, the target stated that “I find adjusting to university life difficult. It is so different from college and being at home”. As in Study 1, and depending on the experimental condition, the target expressed either dejection or agitation in light of the same situation. The dependent variables were assessed as in Study 1 and again included perceived interpersonal regulatory fit [α = .75; *M* = 4.75; *SD* = .93], empathy [α = .85; *M* = 4.95; *SD* = 1.41], willingness to help *[M* = 5.88; *SD* = 1.11], and time spent writing a message of advice to the person in seconds [*M* = 426.55; *SD* = 341.81]. The control variables were also assessed as in Study 1 and again included perceived severity of the situation [*M* = 4.63; *SD* = 1.30], perceived intensity of the negative emotion [*M* = 5.39; *SD* = 1.25], perceived resemblance *[M* = 4.92; *SD* = 1.94], and current positive mood [α = .74; *M* = 4.23, *SD* = .87].

### Results

#### Preliminary analyses

Each control variable was subjected to a 3 [observer regulatory focus: promotion, prevention, control] x 2 [target emotional reaction: dejection, agitation] between-subjects ANOVA. A significant main effect of target reaction for perceived severity, *F*[1,126] = 8.24, *p* = .005, η_p_^2^ = .06, [CI_95%_: .2, 1.09] indicated that the situation was perceived as more severe in the dejection reaction condition [*M* = 4.94, *SD* = 1.17] compared with the agitation reaction condition [*M* = 4.29, *SD* = 1.36]. No other significant effects emerged [all other *F*s*<*2.45, all other *p*s>.12]. Given these unexpected effects, the main analyses below control for perceived severity.

#### Main analyses

Participants’ reported willingness to help, perceived fit, empathy, and time spent on writing a message of advice to the protagonist were subjected to a between-subjects ANCOVA, with the independent variables regulatory focus and target reaction and perceived severity as a covariate. The observer regulatory focus by target reaction interaction was significant for participants’ willingness to help, *F*2,125] = 4.30, *p* = .016, η_p_^2^ = .06, [CI_95%_: 6.08, 6.93]. No other effects emerged [all other *Fs<*2.69, all other *p*s >.07]. Simple effects analyses revealed that there were no effects of target reaction in the prevention focus and the control observer conditions [all *Fs <* .77, all *p*s>.39]. However, in the promotion focus observer condition participants presented with an agitated target were more willing to help [*M* = 6.41, *SD* = .73] compared with those presented with a dejected target [*M* = 5.61, *SD* = 1.10], *F*[1,125] = 8.97, *p* = .003, η_p_^2^ = .07] [CI_95%_: .31, 1.49]. This indicates a misfit effect. Furthermore, participants in the promotion focus condition expressed greater empathy [*M* = 5.28, *SD* = 1.34] compared with those in the control condition [*M* = 4.52, *SD* = 1.43], *F*[2,125] = 4.59, p = .012, η_p_^2^ = .07, [CI_95%_: .16, 1.48]. No significant effects emerged for the other measures [all *F*s<2.25, all *p*s >.14]. [The interaction effect remained significant and no other effects emerged when not including the control variables].

### Discussion

A regulatory focus by target emotional expression interaction emerged that ran counter to our hypothesis. Specifically, for observer promotion focus, a regulatory misfit resulted in greater willingness to help: Participants induced with a promotion focus and presented with agitated target reported greater willingness to help compared with those presented with a dejected target. There were no results for observer prevention focus. Similar to Study 1, participants’ perceptions of the scenarios in terms of perceived severity were not uniform across the target emotional reaction conditions. As mentioned before, in order to ensure scenarios would be perceived equally across target reaction conditions, we ran a pretest of the scenarios used in Studies 3–5 [see S2 Appendix].

A further limitation of this study is the fact that a large number of participants [34%] failed the attention check and were excluded, thus reducing the sample size. The students in the study completed the questionnaire at their own pace, unobserved, and as part of a course requirement, which may have lowered their level of engagement and attention [45]. Consequently, all following studies relied on paid participants.

## Study 3

The same procedures as Study 1 were implemented but a different and pretested scenario of a person suspicious of their partner potentially cheating on them was used in the current study. A different pretested scenario was used in order to mitigate problems of participants perceiving the same situation differently due to different protagonists’ emotional reactions to it.

### Methods

#### Participants and design

Participants’ regulatory focus was measured and they were randomly assigned to one of two experimental target emotional reaction conditions [dejection vs. agitation]. A total of 276 participants completed the study. In addition to the exclusion criteria mentioned above, a total of 10 participants who failed the attention check, probing their attention to instructions and 28 participants who failed the manipulation check, probing the kind of emotional reaction the person expressed in the scenario they had read [dejection vs. agitation], were excluded. The final sample thus comprised 235 participants [152 women, 82 men; *M*_age_ = 33.48, *SD*_age_ = 11.59]. The same variables as in the previous studies were assessed. A sensitivity analysis using G*Power [36] for this study based on the final sample and with an α-level = .05 and a power of 90%, indicated that the minimum size of the effect that could reliably be detected is f^2^ = 0.06.

#### Materials and procedure

The same procedure as Study 1 was used except that a different scenario was presented. Participants filled in the Regulatory Focus Questionnaire [RFQ; [41]], comprising of the promotion focus subscale [α = .72; *M =* 3.37; *SD =* .63] and the prevention focus subscale [α = .81; *M =* 3.24, *SD =* .83]. They were then presented with a scenario describing a person suspicious of their partner potentially being unfaithful. For example, the target stated that “I have noticed that my partner has been spending a lot of time with a particular colleague lately and I can’t help but think there’s something going on between them.” As before, and depending on experimental condition, the target expressed either dejection or agitation in light of the situation equally described to all participants. Participants then reported their perceived interpersonal regulatory fit [α = .83; *M* = 4.69; *SD* = .84], empathy [α = .82; *M* = 4.98; *SD* = 1.04], willingness to help [*M* = 5.17; *SD* = 1.13], and they were invited to write a message of advice to the scenario’s protagonist with their time writing this message being recorded in seconds [*M* = 231.03; *SD* = 204.19]. They also reported their perceived severity of the situation [*M* = 4.40; *SD* = 1.24] and intensity of the negative emotion [*M* = 5.51; *SD* = 1.14], as well as their perceived resemblance to the scenario’s protagonist [*M* = 3.41; *SD* = 1.81]. Their current positive mood was also assessed as before [α = .87; *M* = 4.45, *SD* = 1.01].

### Results

#### Preliminary analyses

Each control variable was regressed on the same predictors and interactions as in Study 1. The stronger participants’ promotion focus was, the less likely they were to report resemblance with the target [*B* = -.14, *SE* = .12, *t* = -2.07, *p* = .041, CI_95%_: -4.76,- .01] and the more likely they were to report overall positive mood [*B* = .39, *SE* = .06, *t* = 6.55, *p* < .001, CI_95%_: .27, .51]. No other significant effects emerged [all other *t*s<1.89, all other *p*s>.06]. Given these unexpected effects, in the main analyses below we control for resemblance and current positive mood. The results indicate that both scenarios were perceived as equally severe.

#### Main analyses

Willingness to help, perceived fit, empathy, and time spent on writing a message of advice to the protagonist were regressed on the same predictors as in the multiple regression above, while controlling for resemblance and current mood. The prevention focus by target reaction interaction effect was significant for willingness to help [*B* = -.16, *SE* = .07, *t* = -2.53, *p* = .012, CI_95%_: -.323, -.04]. Simple slopes analyses [46] revealed that in the agitation reaction condition, the stronger participants’ prevention focus, the more they reported being willing to help [*B* = .24, *SE* = .11, *t* = 2.17, *p* = .031, CI_95%_:.02,.46], but this reversed–though not significantly so–in the dejection reaction condition [*B* = -.12, *SE* = .09, *t* = -1.34, *p*>.18]. This indicates a fit effect, albeit only for prevention, and not promotion, focus. No other effects emerged for willingness to help [all other *t*s<1.25, all *p*s >.21].

Furthermore, the stronger participants’ prevention focus was, the more time they spent writing a message to the target [*B* = .15, *SE* = 13.94, *t* = 2.22, *p* = .027, CI_95%_: 3.61, 58.56]. No other effects were found regarding other measures [all *t*s < 1.63, all *p*s > .10]. [The interaction effect and main effect remained significant and no other effects emerged when not including the control variables].

### Discussion

Partially supporting predictions, participants with a stronger prevention focus and presented with a target expressing agitation rather than dejection reported greater willingness to help this target. However, no such fit effects were found for promotion focus. Furthermore, this fit effect was limited to only one of the prosocial variables considered; it did not emerge for empathy with and time spent on writing a message to the target. Moreover, participants with a stronger prevention focus did not report to perceive greater fit with the target expression of prevention-congruent negative emotions.

The present results are inconsistent with those of Study 2, where a promotion misfit effect occurred such that participants induced with promotion focus reported being more willing to help a target expressing agitation. Although they ultimately do not explain the diverging effects, some differences between these studies are worth pointing out. Firstly, in Study 2 students took part for course credit, a large portion of whom failed a simple attention check, whereas the current study relied on paid Prolific Academic participants, of whom a much lower number failed the same attention check. This clearly suggests better engagement with the current study and, in turn, greater confidence in the present effects. Secondly, the present scenario was pretested. Indeed, the only differences regarding potential control variables were found for regulatory foci, but not for experimental target emotional reaction conditions, indicating that the current scenario ensured that the emotional reaction of the target did not influence the control variables. This also suggests more confidence in the current effects. To potentially generalize effects, Study 4 also relied on a pretested scenario, albeit describing a different situation.

## Study 4

The same procedures as Study 1 and 3 were implemented but a different and pretested scenario of a person struggling to cope with their grandmother’s failing memory was used in the current study, with the aim to generalize effects to a different situation.

### Methods

#### Participants and design

A total of 254 participants completed the study. In addition to the exclusion criteria mentioned above, one participant who failed the attention check and 25 participants who failed the manipulation check probing the nature of the emotional reaction the person expressed in the scenario they read [dejection vs. agitation], were excluded. The final sample thus comprised of 220 participants [151women, 68 men; *M*_age_ = 34.94, *SD*_age_ = 12.09]. Participants’ regulatory focus was measured and they were randomly assigned to one of two experimental target emotional reaction conditions [dejection vs. agitation]. The same variables as in the previous studies were assessed. A sensitivity analysis using G*Power [36] for this study based on the final sample size and with an α-level = .05 and a power of 90%, indicated that the minimum size of the effect that could reliably be detected is f^2^ = 0.06.

#### Materials and procedure

The same procedure as in Studies 1 and 3 was used, but participants were presented with a different pretested scenario. Participants filled in the Regulatory Focus Questionnaire [RFQ; [41]], measuring their promotion focus [α = .73; *M* = 3.28; *SD =* .66] and prevention focus [α = .81; *M* = 3.33; *SD* = .79]. They were then presented with a scenario of a protagonist describing their struggle to cope with their grandmother’s failing memory. For example, the target stated that “It has been very tough for me to witness her memory deteriorating–and her basically “disappearing” before my eyes bit by bit.” As before, and depending on the experimental condition, the target expressed either dejection or agitation in light of the same situation presented to all participants. Participants subsequently reported their perceived overall interpersonal regulatory fit [α = .86; *M* = 5.36; *SD* = .85], empathy [α = .85; *M* = 5.69; *SD* = .98] and willingness to help [*M =* 5.14*; SD =* 1.28] before being invited to write a message of advice to the scenario’s protagonist measured in seconds [*M* = 246.47; *SD* = 204.94]. Also, participants again reported their perceived severity of the situation [*M* = 5.10; *SD* = 1.01], intensity of the negative emotion [*M* = 5.51; *SD* = 1.15] and their perceived resemblance with the protagonist [*M* = 4.04; *SD* = 1.61], as well as their current positive mood [α = .91; *M* = 4.12, *SD* = 1.12].

### Results

#### Preliminary analyses

Each control variable was regressed on the same predictor variables and their interactions as in Studies 1 and 3. Two significant main effect emerged: the stronger participants’ prevention and promotion focus were, the more overall positive mood they reported [promotion: *B* = .32, *SE* = .07, *t* = 5.08, *p* < .001, CI: .22, .49; prevention: *B* = .21, *SE* = .07, *t* = 3.27, *p* = .001, CI_95%_: .09, .39]. There were no other significant effects [all other *t*s>-1.79, all other *p*s>.07]. Given these unexpected effects, in the main analyses below we control for current positive mood.

#### Main analyses

Empathy, perceived fit, willingness to help and time spent on writing a message of advice to the distressed protagonist were regressed on the same predictors and their interactions as in the multiple regression in the previous studies, while controlling for current positive mood. The prevention focus by target emotional reaction interaction was significant for empathy [*B =* .17, *SE* = .07, *t* = 2.45, *p =* .015, CI_95%_: .04, .33]. Simple slopes analysis [46] revealed that the stronger participants’ prevention focus was, the more empathy they reported in the dejection target emotional reaction condition [*B* = .21, *SE* = .09, *t* = 2.29, *p* = .023, CI_95%_: .03, .41]. This reversed, though not significantly so, in the agitation reaction condition [*B* = -.13, *SE* = .12, *t* = -1.23, *p*>.22]. The nature of this interaction indicates a misfit effect. No other significant effects regarding empathy emerged [all *t*s < .95, all *p*s >.34].

Furthermore, the stronger participants’ promotion focus was, the less time they spent on writing a message of advice [*B* = -.19, *SE* = 14.6, *t* = -2.72, *p* = .007, CI_95%_: -68.58, -10.89]. No other significant effects for writing a message of advice or for any other measure emerged [all *t*s<1.73, all *p*s>.09]. [The main interaction effect and main effect remained significant and no other effects emerged when not including the control variables].

### Discussion

The current findings do not support the hypothesis. Participants with a strong prevention focus who were presented with a dejected target were more likely to report greater empathy. This indicates that contrary to the hypothesis, a misfit–rather than a fit–between observers’ regulatory focus and the target’s emotional reaction increased [similar to Study 2 where regulatory focus was manipulated] empathy. The next study aimed to introduce a new appraisal in terms of differences in controllability to strengthen the emotional reactions and similarity in how the scenario would be appraised by observer and target.

## Study 5

In previous studies, participants may not have perceived and appraised the situation similarly as the target because some details about the situation may not have been sufficiently clear. However, similarity in appraisal is important since the appraisal theory of empathy [21] postulates that empathy is particularly likely to occur when an observer appraises a situation similarly as a target. Thus, providing more details regarding the situational appraisal should increase the likelihood of observers and targets appraising the situation similarly.

Specifically, perceived control is an important elements in appraisals, because they result in different emotional reactions to situations [47, 48]. That is, how negative situations are appraised in terms of these variables impacts emotional reactions. Considering control, perceived lack of controllability, or the potential to influence a given situation, has been shown to entail reactions of sadness or dejection, whereas the perception of some degree of controllability of a negative situation more likely results in a reaction of agitation [49]. That is, faced with negative situations and perceiving no or a loss of control, higher levels of dejection can be expected [50]. Similarly, in situations in which one experiences helplessness, dejection is the most common reaction [51].

Therefore, manipulating situational appraisals more concretely regarding control can help ensure that situations are appraised similarly, conveying certain emotional reactions as more plausible, which in turn increases the likelihood of empathy emerging. We consequently aimed to facilitate any potential fit effects by making the emotional reactions appear more plausible and fitting to participants as a result of variations in controllability. For example, in a situation where there is no control [some control], a reaction of dejection [agitation] is expected which will fit with a promotion-focus [prevention], and result in an increase in empathy, willingness to help and prosocial response. Furthermore, adding the controllability factor allows us to investigate an additional explanation for the emotions [through testing the process by interactions] which should influence the interpersonal regulatory fit process [52].

As such, in addition to inducing regulatory focus [as in Study 2], we also manipulated the target’s situational control. In the no control condition, the protagonist had no control over the situation; in the control condition the protagonist had some control to change the situation. We expected that given some changes in control, regulatory focus and target emotional reaction would interact, such that fit effects would most likely emerge for promotion-focused participants reading about a dejected target not having control; and for prevention-focused participants reading about an agitated target having some degree control.

In the current study, participants’ regulatory focus was induced as in Study 2, they were randomly assigned to one of two endings of the same scenario conveying different levels of control [no control vs. control], and they were also randomly assigned target emotional reactions conditions [dejection vs. agitation].

The situation in the scenario was pretested [see S2 Appendix] and entailed a person struggling to adjust to their grandmother’s sudden injury. To assess whether participants’ perceptions of the person in the scenario would impact their responses, social perception items along the dimensions of competency and warmth [53] were also added for exploratory purposes at the end of the study, but they are not considered here.

### Methods

#### Participants and design

A total of 346 participants were randomly assigned to conditions in a 2 [observer regulatory focus: promotion vs. prevention] x 2 [target emotional reaction: dejection vs. agitation] x 2 [controllability of the situation: none vs. some] between-subjects design. The same variables as before, in addition to a manipulation check regarding the controllability of the situation, were assessed. In addition to the exclusion criteria mentioned above, 10 participants who failed the attention check and 60 who failed the manipulation check probing the kind of emotional reaction the person expressed in the scenario they had read [dejection vs. agitation] were excluded. The final sample thus comprised 275 participants [154 women, 117 men; M_age_ = 34.69, SD_age_ = 11.57]. A sensitivity analysis using G*Power [36] for this study based on the final sample size and with an α-level = .05 and a power of 90%, indicated that the minimum size of the effect that could reliably be detected is f = 0.19.

#### Materials and procedure

Participants were randomly assigned to either complete the promotion or the prevention focus induction task as in Study 2 [41]. They were then presented with a pre-tested scenario of a person struggling to cope with their grandmother’s recent injury [see S2 Appendix]. For example, the target stated that “for me it is challenging to see her in this helpless state–I don’t know how long she can continue to live by herself.” Afterwards, participants were randomly assigned to one of two scenario endings: either the target could not do anything to improve the grandmother’s condition [no control] or the target could contribute to improving the grandmother’s condition [some control]. For example, in the no control condition participants read “there is really nothing I can do to change her situation at all” whereas in the some control condition they read “there is at least something I can do to change her situation a bit”. Subsequently, and as in the previous studies, they were randomly assigned to an emotional reaction of the protagonist that took on the form of either dejection or agitation. Participants then reported their perceived interpersonal regulatory fit [α = .78; *M* = 5.41; *SD* = .96], their empathy [α = .86; *M* = 5.88; *SD* = .95], willingness to help [*M* = 5.43; *SD* = 1.2] and wrote a message of advice to the scenario’s protagonist [with their time writing this message being recorded in seconds [*M* = 166.09; *SD* = 156.07].

As potential control measures, participants reported their perceived intensity of the negative emotion [*M* = 5.23; *SD* = 1.44] as in previous studies. Additional control measures, like all other measures assessed on 7-point scales [1 = *not at all* to 7 = *very much*], further explored if the emotional reactions produced differences in perceptions of other aspects as a function of the target’s emotional reaction. These included how natural [*M* = 5.85; *SD* = 1.12], appropriate [*M* = 5.82; *SD* = 1.13], and adequate [*M* = 5.71; *SD* = 1.13] they perceived the protagonist’s reaction [“How natural/appropriate/adequate did the reaction of the person whose grandmother fell seem to you?”]. Participants’ current positive mood was assessed as in the previous studies [α = .89; *M* = 4.39, *SD* = 1.09].

In the current study, participants’ perceived severity of the situation [*M* = 5.26; *SD* = 1.17; “How severe did you find the situation of the person in the text?”; 1 = *not at all* to 7 = *very much*] in this study, served as a manipulation check for the controllability of the situation [i.e., a situation is more severe if it is not controllable]. Finally, as a target emotional reaction manipulation check, participants were asked as in all previous studies to select what kind of emotional response [dejection vs. agitation] the person in the scenario expressed.

### Results

#### Preliminary analyses

Attesting to the success of the controllability manipulation, subjecting participants’ severity ratings to a 2 [observer regulatory focus] x 2 [target emotional reaction] x 2 [controllability] ANOVA, a main effect of controllability on severity was found, *F*[1,267] = 7.2, *p* = .008, η_p_^2^ = .03, [CI_95%_: .09, .64], indicating that in the no control condition the situation was perceived as more severe [*M* = 5.43, *SD* = 1.12] compared with the some degree of control condition [*M* = 5.07, *SD* = 1.19]. This confirms the success of the controllability manipulation.

Each control variables [i.e., severity, intensity, natural, appropriate, adequate, and current mood] was subjected to the same ANOVA as above. A main effect of regulatory focus on intensity emerged, *F*[1,267] = 6.53, *p* = .011, η_p_^2^ = .02, [CI_95%_: .1, .78], indicating that in the promotion condition the target’s negative emotion was perceived as more intense [*M* = 5.47, *SD* = 1.34] compared with the prevention condition [*M* = 5.09, *SD* = 1.53]. A further main effect of target emotional reaction on intensity emerged, *F*[1,67] = 16.23, *p* < .001, η_p_^2^ = .06, [CI_95%_: .35, 1.03] indicating that in the dejection reaction condition the negative emotion was perceived as more intense [*M* = 5.56, *SD* = 1.29] compared with the agitation reaction condition [*M* = 4.91, *SD* = 1.56]. Finally, a significant main effect of controllability on positive mood emerged, *F*[1,267] = 6.91, *p* = .009, η_p_^2^ = .03, [CI_95%_: .08,.61], indicating that in the some degree of control condition participants reported a more positive mood [*M* = 4.55, *SD =* 1.03] compared with the no control condition [*M* = 4.22, *SD* = 1.15]. This mirrors effects obtained for the manipulation check. No other significant effects emerged, all other *F*s*<*3.16, all other *p*s>.08. Given these unexpected effects, in the main analyses below we control for intensity and current positive mood.

#### Main analyses

Participants’ reported willingness to help, perceived fit, empathy, and time spent on writing a message of advice to the distressed target were subjected to a 2 [observer regulatory focus] x 2 [target emotional reaction] x 2 [controllability] ANCOVA controlling for intensity and current positive mood. No significant effects emerged for willingness to help [all *Fs <* .1.27, all *p*s >.26], perceived fit [all *Fs<*3.45, all *p*s >.06], empathy [all *Fs<*2.31, all *p*s >.13] or for time spent on writing the message [all *Fs<*3.35, all *p*s >.07]. [No effects emerged even when not including the control variables].

### Discussion

We expected increased empathy and prosocial responses under conditions of the target expressing dejection and observers having a promotion focus [regulatory fit] in a situation in which the target does not have control to change the situation [compared to when they do have some control]. And we expected similar effects under conditions of the target expressing agitation and observers having a prevention focus [regulatory fit] in a situation in which the target does have some control to change the situation [compared to when they do not]. Contrary to these predictions, the results revealed no significant effects.

## Meta-analysis of Studies 1–5

Five studies tested our hypothesis. These studies differed in terms of scenarios used and populations of respondents, as well as in terms of whether regulatory focus was measured [Studies 1, 3 & 4] or induced [Study 2 & 5]. Overall, they provided inconsistent results. Therefore, a meta-analysis simultaneously taking into account all effects across studies seemed warranted in order to gauge the nature of a potential effect emerging across studies. To compute an overall test for the predicted interaction effect, of observer regulatory focus [both chronic and induced] and target emotional reaction condition [dejection vs. agitation] on the two variables most pertinent to our predictions and to which some effect emerged across studies, namely empathy and willingness to help, we conducted a meta-analysis separately for each of the measures. To better be able to compare effects across studies, for Studies 1 and 2, the control conditions were not included; for Study 5 the controllability factor was not included.

All effect sizes were transformed into Pearson’s *r* [see Table 1] and the Sidik-Jonkman method was applied [54] with a random effects model [55]. Across the studies, the following result emerged for willingness to help: *r* = 0.05, *p* = 0.11, CI_95%_ [0.14062; -0.0336]. With zero included in the confidence interval, this clearly indicates no significant effect. Similarly, for empathy no significant result across studies emerged: *r* = -0.04, *p* = 0.19, CI_95%_ [0.04308; -0.1139]. In sum, the two meta-analyses revealed no significant effects of interpersonal regulatory fit on willingness to help or empathy.

**Table 1:** Sample sizes and effect sizes regarding willingness to help and empathy in all studies.

Study	Sample size for willingness to help	Effect size (Pearson’s *r*) Willingness to help	Sample Size Empathy	Effect size Empathy
1	179	-0.004	179	-0.016
2	94	-0.221	91	0.022
3	238	0.093	241	0.045
4	220	-0.075	220	-0.098
5	268	0.089	268	0.015

## General discussion

This set of studies tested whether observers perceiving a target expressing a negative emotion in response to a distressing situation would report greater perceived fit with the target, willingness to help, empathy, and show more prosocial behaviour under conditions of interpersonal regulatory fit. Interpersonal regulatory fit occurred for observer promotion focused-target expressing dejection or observer prevention focused-target expressing agitation compared to cases of misfit that involved observer promotion-focused-target expressing agitation or observer prevention-focused-target expressing dejection. Across five studies, we did not find support for this prediction. Moreover, the pattern of results was inconsistent and an internal meta-analysis indicated that overall effects did not differ from zero.

The mixed effects are arguably rather difficult to interpret and we can only speculate on them. As we detail in much length below, against the background of pervious work that largely considered interpersonal regulatory fit in the context of goal pursuit the null effects–as well as the absence of effects indicated by the meta-analysis–the current studies can be understood as an indication that merely providing foci congruent emotions might not suffice as an indicator of another person sharing [or not] one’s regulatory focus, nor for initiating empathy or prosocial tendencies. At the same time, the misfit effects present in Studies 2 and 4 could be seen as in indicator that people are especially reactive to others that display emotional distress running *counter* to what they would assume others to feel based on their foci-related appraisal of the situation [though to the best of our knowledge no such effects have been previously reported]. Contrary, the prevention fit effect in Study 3, in which the scenario involved a partner potentially cheating, might have been sustained by the situation aligning more strongly with security and loss concerns more present in a prevention compared to a promotion focus, suggesting that considering concerns at stake might contribute to potential fit effects. We believe that overall the safest conclusion to be drawn should based on our meta-analysis across studies: Countrary to previous work [34], the present findings do not support the notion that similarity in terms of regulatory focus entails empathy, at least not with the paradigms explored here.

Positive effects of interpersonal regulatory fit have been reliably demonstrated regarding various outcome measures, such as followers evaluating leaders with a similar regulatory focus more positively [27], people being more motivated by role models of the same regulatory focus [56], and supervisees experiencing greater commitment and better relationship quality with supervisors in the workplace when they share the same regulatory focus [30]. A crucial difference compared to the present work is that in these studies the regulatory focus of the to-be-considered targets was conveyed rather clearly through their regulatory concerns [e.g., goals], whereas in the current work targets’ regulatory focus was conveyed merely through their emotional reaction to distressing situations. For example, Hamstra, Van Yperen, Wisse & Sassenberg [27] conveyed targets’ regulatory focus through a description of their goals in the form of a job application letter that either conveyed promotion concerns [i.e., the target expressed ‘I have a strong desire for a position in which I am challenged’] or prevention-concerns [i.e., the target expressed ‘I have a strong desire for a position with a lot of responsibility’]. In another study, Righetti, Finkenauer, & Rusbult [11] assessed the effect of partner regulatory focus on participants’ goal-pursuits. Participants reported on the regulatory focus of their partner with questions such as: “He or she often thinks about how he or she will achieve personal or professional success” for the promotion scale or “He or she frequently thinks about how he or she can prevent failures in his or her life” for the prevention scale. Contrary, the present work expected that participants would be able to have a notion of targets’ regulatory focus based on the limited information conveyed by their different emotional reactions; this was the only indicator of the target’s regulatory focus that was available to participants. Having at least a vague notion of targets’ regulatory concerns might have consequently been more difficult in the current compared to previous research, which in a much more straight-forward way portrayed targets’ regulatory focus. In turn, they might have created clearer experience of interpersonal regulatory [mis]fit in participants. Future research might thus be advised to add elements of the distressed targets’ goal-pursuits and regulatory concerns to the scenarios, in addition to describing different qualities of the distressed emotional reactions.

A further element future research might take into account when seeking to enrich scenarios concerns information on goal-pursuit failure. Indeed, previous studies assessing the relationship between regulatory focus and emotional reactions considered different emotions of different intensity in reaction to failure or success in goal-pursuit [8, 10, 57–59]. For example, Idson, Liberman, & Higgins [57] examined how participants with different regulatory foci would feel in response to a negative outcome of failure or a positive outcome of success. Thus, unlike the current work these studies did not assess emotional reactions resulting from distressing situations *per se*, but rather in reaction to failure [or success] in *goal-pursuit*. As such, observers might more easily perceive dejection, respectively agitation, conveying a regulatory orientation when these emotional reactions are a response to unsuccessful goal-pursuit. Future research might thus be advised to consider enriching scenarios by including goal-pursuit failure information.

Importantly, emotions are immediate short-term experiences, describing behaviour whereas goals, by comparison, are considered as a longer lasting or more stable part of the self [60]. Interpersonal regulatory fit effects should be stronger based on matches between selves or attributes compared to matches between a target’s behaviour and an observers’ attributes. As such, a regulatory fit effect may be expected based on target attributes but not based on short-lived target behaviour [such as emotions]. Success or failure in focus congruent goal pursuit [based on focus congruent concerns] is assumed to lead to different emotional reactions [10]. However, emotions–in turn–do not necessarily convey such regulatory concerns. As such, by merely conveying different emotions the foci of targets’ was not necessarily clear to participants. This suggests that relying only on emotional reactions in the field of regulatory focus theory generally, or on regulatory fit more specifically, might not suffice. It also suggests the need to convey targets’ goals, strategies for achieving them, and/or regulatory concerns and priorities–more stable aspects of the self than emotions and more clear indicators of regulatory inclinations.

To the best of our knowledge, there is only one study which presented targets’ emotional reactions to distressing situations [rather than to failure or success in goal-pursuit] in the context of interpersonal regulatory [mis]fit [34]. This study assessed the effect of [dis]similarity in terms of self-discrepancies, that is discrepancies between people’s actual self and their ideal self [what they think they want to be] or ought self [what they think they should be] on empathy. Much as in the present work, observer-target [dis]similarities were conveyed through emotional reactions of targets to distressing situations. This study found that observers sharing [vs. not sharing] the same self-discrepancy with targets perceived larger similarity to the target and reported stronger empathic responses.

Although the present work in part builds on this research, a number of differences between the current work and that of Houston [34] are important to note, as they are potentially informative to why the current work did not find similar results regarding regulatory focus. First, Houston included only participants with extreme scores: participants either had a very high or a very low ideal or ought self-discrepancies, respectively. Focusing on such extreme cases increases both the likelihood of finding effects, as well as their magnitude. By contrast, the present work considered normal distributions of participants’ promotion and prevention foci–a desirable feature in terms of parametric testing, but that at the same time renders potential effects less strong. Second, in the scenarios employed by Houston, participants saw videos of targets conveying their emotions along with differences in terms of gestures, voice tonality, and facial expressions. Contrary, the current work relied on participants merely reading about a target describing emotional reactions. Thus, the current work provided less information about the target, which may have resulted in diminishing notions of interpersonal regulatory fit. Third, apart from their emotional reactions targets additionally expressed their motivational state with regard to failing to meet their ideal or ought self-guide. This dovetails with the studies above conveying regulatory focus more clearly by providing information on success and failure in goal-pursuit. Overall, Houston’s procedures may thus have allowed for a more realistic impression of the target, created a more impactful experience for participants, and left them with a clearer notion of targets’ shared or unshared self-discrepancies, in turn enhancing [mis]fit effects. Future research on interpersonal regulatory fit in the realm of emotional reactions would thus be advised to introduce more explicit conditions of targets’ situations, reactions, and goal-pursuit, thus conveying targets’ regulatory focus more clearly.

Contrary to the present work, in most previous studies on interpersonal regulatory fit outcomes variables were comparatively less dependent on participants’ emotional responses to targets, and instead more strongly pertained to goal-pursuit concerns. In other words, the bulk of previous work investigated interpersonal regulatory fit in the context of goal-pursuit. For example, outcome variables included turnover intentions [31], expending greater effort at work [32], motivational benefits in goal-pursuit [11] or motivational gains [56]. On the other hand, emotions–the outcome of [un]successful goal pursuit–received attention at the individual [e.g., 10] but not at the interpersonal level [but see 34, for an exception]. It stands to reason that because emotions are the result of [un]successful goal pursuit, interpersonal self-regulatory–and other–considerations become less relevant.

Conveying targets’ regulatory focus with more clarity could be achieved by including behavioural strategies or plans to be undertaken in line with either foci. For example, in a distressing situation where partner infidelity is suspected [such as in Study 3], a promotion-focused target might state their behavioural plans to eagerly talk to their partner about the issue in order to ensure re-connecting with them. Instead, a prevention-focused target might state that they will be especially vigilant when talking to their partner about the issue in order to avoid further increasing their interpersonal distance.

We also recommend changes in methodological issues when designing similar future studies. These include exploring and reassessing how targets’ distress is presented and the use of online data collection. In the present work, we only used distressing scenarios or vignettes to present presumably [mis]fitting social targets. However, the adequacy of using vignettes to investigate certain outcome variables, such as empathy, may be especially problematic. According to the appraisal theory of empathy [21], a crucial aspect for empathy to emerge is appraising a situation in a similar way as others [and thus in the present studies as the targets in the scenarios]. This, however, often requires having sufficient information about the situation at stake. Learning about targets’ situations indirectly and to a limited degree [such as is the case of reading a vignette] is likely to entail observers having less and/or insufficient information about the situation, compared to directly encountering the targets’ situations in vivo or at least in a video. In other words, short vignettes might arguably render rather difficult understanding a given situation well enough to appraise it accurately. Accordingly, Hughes and Huby [61] suggest the use of images and videos in conjunction with texts in experiments. This can serve to engage more than one of participants’ senses and offers a more involving depiction of targets’ distress. Moreover, they suggest that videos and images might capture and maintain participants’ attention, enhance their motivation, and provide them with a sense of realism–compared to simply reading a text. Future studies may thus benefit from incorporating these formats.

A further methodological issue concerns the question whether online studies are suited to investigate the present research question, and interpersonal regulatory fit more generally. Importantly, most research on interpersonal regulatory fit has been conducted offline, in lab settings, rather than online [e.g., 24, 28, 56, but see Hamstra et al., 27]. Inherent in interpersonal regulatory fit is an element of *interpersonal* relatedness or connection and that might be especially difficult to create in online settings [62]: People might very well be less likely to experience fit with another person in such a rather impersonal format. The present research was based on the expectation that participants relate to and experience interpersonal processes such as fit with a fictional target online. Whilst participants arguably are able to form impressions of targets online, this may less likely entail interpersonal experiences and consequences such as fit or empathy, compared to laboratory settings. In short, interpersonal experience and ensuing emotional reactions might be more challenging to achieve online.

Similarities between observers and distressed targets regarding various attributes have been shown to lead to greater empathy and helping [e.g., [5, 6]. However, the similarities considered by previous work largely relied on attributes that can be consciously and easily perceived and taken into account by observers. A crucial difference between similarity of attributes and similarity of regulatory focus is that regarding the latter individuals are unlikely to be explicitly aware of indeed sharing this similarity. This further highlights the difficulties in documenting effects of regulatory [mis]fit and the need to make targets’ regulatory focus of targets clearer to participant observers.

We also acknowledge the issue of power is a critical aspect in some of our studies. Notably, and particularly for Studies 1 and 2, a number of participants failed the attention and manipulation of emotional reaction check, rendering these studies less powered than would have been desirable. Nevertheless, the sensitivity analyses overall showed that Studies 3, 4, and 5 were not as underpowered. Moreover, the internal meta-analysis based on all data we collected on this research question was well powered and did not detect an overall effect.

The current work investigated whether a fit between observers’ regulatory focus and targets’ emotional reactions expressed after various distressing situations would increase their likelihood to express empathy and to prosocially respond to the target. Because of inconsistent findings and contradictory results, conclusions regarding this hypothesis cannot be drawn. Nonetheless, the current work is informative insofar as it highlighted the technical and practical challenges that arise when designing a situation intended to elicit empathy based on sharing subtle similar features such as self-regulatory orientations. It remains that the hurdles of this endeavour can serve as a guide and provides a stepping stone for future studies attempting to clarify the potential impact of interpersonal regulatory fit on empathy and willingness to help.

## Supporting information

S1 Appendix Scenario of Study 1. (DOCX)

S2 Appendix Pre-test of five scenarios. (ODT)

S3 Appendix Scenario of Study 2. (ODT)

S4 Appendix Scenario of Study 3. (ODT)

S5 Appendix Scenario of Study 4. (ODT)

S6 Appendix Scenario of Study 5. (ODT)
