# Supplementary material for: Testing the impact of interpersonal regulatory fit on empathy, helping intentions, and prosocial behaviour
Source: PLoS One. 2022 Jul 18;17(7):e0270462. doi: 10.1371/journal.pone.0270462 (PMC9292111; doi:10.1371/journal.pone.0270462)
Supplement: S1 Appendix — (DOCX) [file pone.0270462.s001.docx]

S1 Appendix A

Scenario

I have just recently started a new job at a large multinational firm. To be honest, I am not really enjoying it so far, apart from a few tasks which are interesting. It’s been sort of a really tough transition for me because I am used to a smaller and more tight-knit working environment, with much clearer guidelines and tasks. I do not feel appreciated as in my previous job and I am feeling much less motivated as a result. Sometimes I think I should not have changed jobs, but it’s too late now.

*Agitated reaction*

I am actually feeling very nervous about this transition. It’s been really hard to calm down and focus because I feel so tense. Also, I’m really worried about getting along with my colleagues – and afraid of not performing up to expected standards. I find this very difficult and know I should be making more of an effort to stay calm. But I am feeling just so stressed out about it, especially because I know this is the most crucial time to make an impact for my future career. I’m hoping something changes soon – right now I feel terribly anxious.

*Dejected reaction*

I am actually feeling very sad about this transition. It’s been really hard to stay positive and focus because I feel so unhappy. Also, I’m really discouraged about getting along with my colleagues – and sad about not performing up to expected standards. I find this very difficult and know I should be making more of an effort to stay positive. But I am feeling just so down about it, especially because I know this is the most crucial time to make an impact for my future career. I’m hoping something changes soon – right now I feel terribly depressed.
