# Supplementary material for: Testing the impact of interpersonal regulatory fit on empathy, helping intentions, and prosocial behaviour
Source: PLoS One. 2022 Jul 18;17(7):e0270462. doi: 10.1371/journal.pone.0270462 (PMC9292111; doi:10.1371/journal.pone.0270462)
Supplement: Supplementary file 2 [file pone.0270462.s002.odt]

S2 Appendix B

Pre-test for scenarios

Five scenarios were pretested to ensure they would elicit similar reactions on relevant control variables across the dejection and agitation reaction conditions. On Prolific Academic, a total of 35 participants (15 males, 20 females; Mage=31.71, SDage=11.25) were recruited and took part online in a 5-minute study. They were presented with the five scenarios, and for each scenario two emotional target reactions were presented. Whilst the order of the scenarios was not varied, the emotional reaction was counterbalanced. In other words, this pretest had a 5 (type of scenario) by 2 (emotional reaction) within-subjects design. Participants were asked to rate each emotional response on a 7-point scale (1= not at all to 7= very much) on the following variables: severity (“How severe did you find the situation of the person in the text?”), intensity (“How intense was the negative emotion the person was expressing in the text?”), natural (“How natural did the reaction of the person in the text seem?”), appropriateness (“How appropriate did the reaction of the person in the text seem?”), and adequacy (“How adequate did the reaction of the person in the text seem?”).

Results 

Paired-samples t-tests were conducted to compare judgments on the control variables across target reactions for each scenario[^1]. Significant or marginal differences between the two target reactions emerged for only two of the scenarios presented. For scenario 1 (unemployment scenario), the agitation reaction (M=5.86; SD=1.03) was judged as significantly more appropriate than the dejection reaction (M=5.43; SD=1.34), t(34)=2.22, p =.03. For scenario 5 (Kidney failure scenario), the dejection reaction (M=6.14; SD=1.17) was judged as significantly more intense than the agitation reaction (M=5.80; SD=1.11). For this scenario, a marginal effect was also detected regarding the reaction being natural, with the dejection reaction (M=6.31; SD=.96) judged as slightly more natural than the agitation reaction (M=5.91; SD=1.44), t (34) =-1.98, p=.05. However, no significant differences emerged for the remaining three scenarios. That is, for scenario 2, 3, and 4 no differences on all the assessed control variables emerged (all ts>-1.61, all ps>.12). Notably, however, in scenario 3 (motor neuron disease), the situation was judged as highly severe for both the agitation reaction (M= 6.40; SD=1.06) and for the dejection reaction (M=6.20; SD=1.12) and therefore it was not considered for future studies. Scenario 2 (cheating partner) and scenario 4 (grandmother’s failing memory) seem to be the most appropriate scenarios for the purposes of this research as they showed similar means on severity (scenario 2: agitation M=4.71; SD=1.36; dejection: M=4.94; SD=1.33; scenario 5: agitation M=5.94; SD=.68; dejection: M=5.91; SD=.92). 

The scenario for study 5 was pretested to ensure it would elicit similar reactions on relevant control variables across the dejection and agitation reaction conditions, as well as across controllability (control versus no control conditions). On Prolific Academic, a total of 62 participants (14 males, 48 females; Mage=36.47, SDage=10.81) were recruited and took part online in a 5-minute study. All participants were presented with the same scenario of a grandchild coping with their grandmother’s injury. Controllability of the situation was manipulated through presenting two different endings of the same scenario. Participants were randomly assigned to one of two different endings of the scenario, one where there was nothing the grandchild could do to improve the grandmother’s condition (no control) or one where the grandchild could help improve the grandmother’s condition (control). Afterwards, all participants were presented with the same two emotional target reactions (agitation versus dejection; order was counterbalanced). Finally, as a manipulation check, they were asked to recall if the person in the scenario had no control or some control over their situation. In other words, this pretest had a 2 (controllability; between participants) by 2 (emotional reaction; within participants) mixed design. After participants who failed the manipulation check were excluded, a total of 36 participants remained.[^2] 

Participants were asked to rate each emotional response on a 7-point scale (1= not at all to 7= very much) regarding the following variables: severity (“How severe did you find the situation of the person in the text?”, M=5.49; SD=1.05), intensity (“How intense was the negative emotion the person was expressing in the text?”, M=5.59; SD=.95), natural (“How natural did the reaction of the person in the text seem?”, M=5.94; SD=.84), appropriateness (“How appropriate did the reaction of the person in the text seem?”, M=5.71; SD=.88), adequacy (“How adequate did the reaction of the person in the text seem?”, M=5.71; SD=.89), gentle (“To what extent does the person whose grandmother fell seem like a gentle person to you?”, (M=5.69; SD=.96), warm (“To what extent does the person whose grandmother fell seem like a warm person to you?” M=5.78; SD=1.08), self-confident (“To what extent does the person whose grandmother fell seem like a self-confident person to you?” M=4.38; SD=1.27) and competent (“To what extent does the person whose grandmother fell seem like a competent person to you?” M=5.43; SD=1.04). Participants were then debriefed and remunerated.

Results and Discussion

A 2 (emotional reaction; within participants) x 2 (controllability; between participants) mixed ANOVA was run for each of the variables above. No significant differences emerged on the main and the interaction effects on all the variables (all Fs<4.14; all ps>.06). As such, the scenario was deemed appropriate to use for study 5. 

[^1]: A 5 (five scenarios) x 2 (agitation versus dejection) within subjects ANOVA was also run separately for severity, intensity, natural, appropriateness, and adequacy to examine differences on scenario, target reaction and the interaction of scenario by target reaction. No effect of target reaction and interaction of target reaction by scenario on all measures emerged (all Fs<2.62, all Ps>.06).
[^2]: A large number of participants failed the manipulation check: “Did the person whose grandma fell have some control over changing the grandma’s situation to the better?” with the two available options: the person has some control or the person has no control. Many participants chose ‘some’ control versus no control, this is perhaps due to the wording used, with ‘some’ appearing to be the less extreme choice. This manipulation check was not used in Study 5.
