# Supplementary material for: Testing the impact of interpersonal regulatory fit on empathy, helping intentions, and prosocial behaviour
Source: PLoS One. 2022 Jul 18;17(7):e0270462. doi: 10.1371/journal.pone.0270462 (PMC9292111; doi:10.1371/journal.pone.0270462)
Supplement: Supplementary file 3 [file pone.0270462.s003.odt]

S3 Appendix C

I’m a first year student at Roehampton University. I just recently started here. To be honest, I am not really enjoying it so far, apart from certain classes which are interesting. It’s been sort of a really tough first semester for me because I find adjusting to university life difficult. It is so different from college and being at home. Sometimes I think I should not have come to university.

Agitated reaction 

 I am actually feeling very nervous about living away from home. It’s been really hard to calm down and focus because I feel so tense. Also, I’m really worried about making new friends – and afraid of losing touch with my friends back home. I find this very difficult and know I should be making more of an effort to stay calm. But I am feeling just so stressed out about it, especially because I know this is the most crucial time to make friends. I’m hoping something changes soon – right now I feel terribly anxious.

Dejected reaction

I am actually feeling very sad about living away from home. It’s been really hard to stay positive and focus because I feel so unhappy. Also, I’m really discouraged about making new friends – and sad about losing touch with my friends back home. I find this very difficult and know I should be making more of an effort to stay positive. But I am feeling just so down about it, especially because I know this is the most crucial time to make friends. I’m hoping something changes soon – right now I feel terribly depressed.
