# Supplementary material for: Testing the impact of interpersonal regulatory fit on empathy, helping intentions, and prosocial behaviour
Source: PLoS One. 2022 Jul 18;17(7):e0270462. doi: 10.1371/journal.pone.0270462 (PMC9292111; doi:10.1371/journal.pone.0270462)
Supplement: Supplementary file 4 [file pone.0270462.s004.odt]

S4 Appendix D

Scenario

I have been with my partner for almost 5 rather happy years. But in the last few months our relationship has been strained and I am sensing some growing distance between us. We have not been spending as much time together as we used to because my partner doesn’t seem to be interested in that. There’s also been a change in my partner’s behaviour towards me, and I think my partner is confiding in and relying on work colleagues and friends – rather than me – more and more. Even worse, I have noticed that my partner has been spending a lot of time with a particular colleague lately and I can’t help but think there’s something going on between them. I also often catch myself thinking that my partner has disengaged from our relationship and doesn’t want to communicate with me. I am in a very difficult place right now.

Agitated reaction 

I am actually feeling very anxious about the state of our relationship. It’s been really hard to calm down and address my situation because I feel so tense. I’m very worried about the growing distance between us. I find this very difficult and it is hard for me to stay calm. I feel afraid when thinking about bringing this up with my partner – and I also feel stressed, especially because I don’t really understand what is happening between us. It just agitates me. I feel terribly anxious.  

Dejected reaction

I am actually feeling very discouraged about the state of our relationship. It’s been really hard to stay optimistic and address my situation because I feel so unhappy. I’m very sad about the growing distance between us. I find this very difficult and it is hard for me to stay positive. I feel depressed when thinking about bringing this up with my partner – and I also feel down, especially because I don’t really understand what is happening between us. It just saddens me. I feel terribly depressed.
