# Supplementary material for: Testing the impact of interpersonal regulatory fit on empathy, helping intentions, and prosocial behaviour
Source: PLoS One. 2022 Jul 18;17(7):e0270462. doi: 10.1371/journal.pone.0270462 (PMC9292111; doi:10.1371/journal.pone.0270462)
Supplement: Supplementary file 5 [file pone.0270462.s005.odt]

S5 Appendix E

Scenario

I have always had a very close relationship with my grandmother and visit her frequently. She has always been someone I could rely on and get good advice from regarding many aspects of my life. She’s a great person and I care about her a lot. Recently, she has started to show some memory loss. It all began with minor and seemingly insignificant things, like confusing dates and medication. But this steadily developed into more serious forgetfulness. She also started to be disoriented at times, not knowing where she was. Last week, it was difficult to hear her speak about events that I know never took place. It has been very tough for me to witness her memory deteriorating – and her basically “disappearing” before my eyes bit by bit. I try to visit as often as I used to, but every time it is getting more and more difficult for me to watch her worsen like this. I don’t know how long she can continue to live by herself with just some help from caretakers checking on her. I assume I will have to take on a good share of caretaker responsibilities as neither she nor my family have lots of resources. I'm not sure how I will manage that.

Agitated reaction 

I am actually feeling very anxious about her condition. Since she started losing her memory, it’s been really hard to calm down when I am around her and for me to live a normal life because I feel so tense. Also, seeing her getting worse really worries me – and I feel afraid of losing her further with time. I find this very difficult to cope with and it is hard for me to stay calm in my personal life. I am feeling just so stressed out about it all, also because I know there is not much I can do. I feel terribly anxious.  

Dejected reaction

I am actually feeling very depressed about her condition. Since she started losing her memory, it’s been really hard to stay positive when I am around her and for me to live a normal life because I feel so down. Also, seeing her getting worse really discourages me – and I feel sad about losing her further with time. I find this very difficult to cope with and it is hard for me to stay upbeat in my personal life. I am feeling just so discouraged about it all, also because I know there is not much I can do. I feel terribly depressed.
