# Supplementary material for: Testing the impact of interpersonal regulatory fit on empathy, helping intentions, and prosocial behaviour
Source: PLoS One. 2022 Jul 18;17(7):e0270462. doi: 10.1371/journal.pone.0270462 (PMC9292111; doi:10.1371/journal.pone.0270462)
Supplement: Supplementary file 6 [file pone.0270462.s006.odt]

S6 Appendix F

Scenario

No Control

I’ve always had a very close relationship with my grandmother and visit her regularly. She is an important person in my life and someone I could truly rely on. Recently, she had a very serious fall and had to be rushed to the hospital. She was very badly injured and underwent immediate surgery on her leg. After the surgery, we were told she would just need some time as a recovery period. Unfortunately, it transpired she had very limited range of motion in her leg and the situation appeared to be more serious than we first thought. For the past month, I have had to take time off work for two weeks to keep a close eye on her. I live near my grandmother and there is no one else in my family able to help out. It has become increasingly difficult for her to manage even basic tasks on her own. And for me it is challenging to see her in this helpless state – I don’t know how long she can continue to live by herself. 

At her last appointment, the doctor said that her condition is unstable and will very likely worsen. The doctor also made it clear that further operations or physiotherapy will not make a difference at all. I assume I will have to take on a good share of caretaker responsibilities as neither she nor my family has lots of financial resources to pay for care. So there is really nothing I can do to change her situation at all. 

Control

I’ve always had a very close relationship with my grandmother and visit her regularly. She is an important person in my life and someone I could truly rely on. Recently, she had a very serious fall and had to be rushed to the hospital. She was very badly injured and underwent immediate surgery on her leg. After the surgery, we were told she would just need some time as a recovery period. Unfortunately, it transpired she had very limited range of motion in her leg and the situation appeared to be more serious than we first thought. For the past month, I have had to take time off work for two weeks to keep a close eye on her. I live near my grandmother and there is no one else in my family able to help out. It has been increasingly difficult for her to manage even basic activities on her own. And for me it is challenging to see her in this helpless state – I don’t know how long she can continue to live by herself.  

At her last appointment, however, the doctor said that with enough physiotherapy her condition will become more stable and will not worsen if she gets daily and time-intensive physiotherapy. I am able to learn the exercises from the physiotherapist myself and help her with them. I assume I will have to take on a good share of caretaker responsibilities as neither she nor my family has lots of financial resources to pay for care. So there is at least something I can do to change her situation a bit.

Dejection

I am actually feeling very depressed about her condition. Ever since her fall, it’s been really hard to stay positive when I am around her and for me to live a normal life because I feel so down. Seeing her struggle really discourages me – I feel sad about seeing her suffer. I find this very difficult to cope with and it is hard for me to stay upbeat in my personal life. I am feeling just so discouraged about it all. I feel terribly depressed.

Agitation

I am actually feeling very anxious about her condition. Ever since her fall, it’s been really hard to calm down when I am around her and for me to live a normal life because I feel so tense. Seeing her struggle really worries me – I feel afraid when seeing her suffer. I find this very difficult to cope with and it is hard for me to stay calm in my personal life. I am feeling just so stressed out about it all. I feel terribly anxious.
